# Pancreatic Ultrasound Features at Diagnosis of Type 1 Diabetes: Age-Related Differences in Children

**DOI:** 10.3390/jcm14217490

**Published:** 2025-10-23

**Authors:** Emre Özer, Sefa Tığrak, Ayşe Seçil Ekşioğlu, Pınar Kocaay, Abdurrahman Bitkay, Keziban Toksoy Adıgüzel, Mehmet Boyraz, Fatih Gürbüz

**Affiliations:** 1 Department of Pediatric Endocrinology, Ankara Bilkent City Hospital, Ankara 06800, Türkiye; pinarbozdemir@yahoo.com (P.K.); abdurrahman.bitkay@saglik.gov.tr (A.B.); drkezibantoksoy@outlook.com (K.T.A.); boyrazoglu@gmail.com (M.B.); fggurbuz@yahoo.com (F.G.); 2 Department of Pediatric Radiology, Ankara Bilkent City Hospital, Ankara 06800, Türkiye; sefatigrak@yahoo.com (S.T.); aseksioglu@aybu.edu.tr (A.S.E.); 3 Department of Radiology, Medical School, Ankara Yıldırım Beyazıt University, Ankara 06800, Türkiye; 4 Department of Pediatric Endocrinology, Medical School, Ankara Yıldırım Beyazıt University, Ankara 06800, Türkiye

**Keywords:** pancreatic echogenicity, pancreatic size, T1D endotypes, type 1 diabetes mellitus, ultrasonography

## Abstract

**Background/Objectives:** To evaluate pancreatic size and echogenicity using ultrasonography in newly diagnosed pediatric Type 1 Diabetes Mellitus patients within five days of diagnosis, and compare early childhood (<7 years) and adolescent (≥13 years) endotypes with clinical and laboratory findings. **Methods:** This prospective, cross-sectional, case–control study included 69 pediatric patients with newly diagnosed type 1 diabetes mellitus, aged 1–18 years, and 78 age- and sex-matched healthy controls. Patients with chronic conditions (e.g., pancreatitis or cystic fibrosis), other forms of diabetes, or medications affecting glucose metabolism were excluded. Ultrasonography was performed within five days of diagnosis, after metabolic stabilization, to assess pancreatic dimensions and echogenicity. Laboratory analyses included measurements of C-peptide, HbA1c, and autoantibodies (anti-GAD, islet cell antibody, and insulin antibody). **Results:** Pancreatic dimensions were significantly smaller in type 1 diabetes mellitus patients (*p* < 0.001), with greater reductions in adolescents (head: 21%, body: 26.7%) vs. young children (head: 14.4%, body: 15.5%). Isoechoic pancreases were more common in young patients (80% vs. 40.9%; *p* = 0.033). C-peptide and HgbA1c were higher in adolescents (*p* < 0.05), with no echogenicity–autoantibody association. **Conclusions:** This first early-post-diagnosis ultrasonography study reveals age-specific pancreatic atrophy and echogenicity changes in children, more severe in adolescents, reflecting type 1 diabetes mellitus endotypes. Ultrasonography offers a practical noninvasive tool for early detection and endotype stratification, informing personalized diabetes care.

## 1. Introduction

Type 1 diabetes mellitus (T1DM) is a heterogeneous autoimmune disorder characterized by the progressive destruction of pancreatic beta cells, leading to absolute insulin deficiency [1]. Globally, its incidence in children varies from 3.9 to 57 per 100,000, with rising trends in many regions [2]. While often presenting with acute symptoms of hyperglycemia, T1DM can also be detected incidentally through screening. Lifelong insulin therapy, combined with lifestyle modifications, is essential for management; however, suboptimal control increases risks of microvascular and macrovascular complications, including cardiovascular disease, neuropathy, and nephropathy [3,4]. The pathogenesis of T1DM involves a complex interplay of genetic susceptibility, environmental triggers, and dysregulated immune responses, resulting in distinct disease processes [5]. As the disease evolves, the pancreas undergoes structural remodeling, including reduced volume and altered parenchymal integrity, which may precede clinical onset [6]. In pediatric T1DM, emerging evidence supports two primary immunological endotypes based on age at diagnosis: the early childhood endotype (T1DE1, onset < 7 years), marked by rapid, aggressive inflammation, and the adolescent endotype (T1DE2, onset ≥ 13 years), characterized by slower, protracted beta-cell loss. Children aged 7–12 years often display intermediate features. These endotypes reflect underlying heterogeneity in immune profiles, autoantibody patterns, and residual beta-cell function, with implications for personalized prevention and therapy [7]. Although magnetic resonance imaging (MRI) and computed tomography (CT) remain gold standards for pancreatic visualization, ultrasonography (USG) offers a non-invasive, accessible alternative for evaluating organ size, echogenicity, and heterogeneity, key markers of exocrine and endocrine dysfunction [8]. Prior studies have documented pancreatic atrophy in established T1DM, but data on early structural changes at diagnosis, particularly via USG, are limited. Moreover, age-related differences in these alterations and their alignment with endotypes remain underexplored.

This prospective study is the first to investigate pancreatic morphology using USG within five days of T1DM diagnosis in pediatric patients, following metabolic stabilization. We aimed to quantify structural changes in pancreatic size and echogenicity compared to healthy controls and to explore age-specific differences between early childhood- and adolescent-onset groups, alongside clinical and laboratory parameters. Overall by linking these findings to T1DM endotypes, this work also aimed to provide novel insights into disease heterogeneity, potentially guiding non-invasive monitoring and tailored therapeutic strategies.

## 2. Subjects, Materials and Methods

This study was designed as a prospective, cross-sectional, case–control study, including 69 pediatric patients aged 1–18 years who were newly diagnosed with type 1 T1DM, and 78 healthy children as the control group. Patients with chronic conditions (e.g., pancreatitis, cystic fibrosis), other forms of childhood diabetes (such as type 2 diabetes mellitus or maturity-onset diabetes of the young), or those receiving medications known to affect glucose metabolism (e.g., steroids, diazoxide) were excluded. The control group consisted of age- and sex-matched healthy children without chronic illnesses, metabolic syndrome, or abnormal glucose metabolism (based on clinical history and physical examination). All participants were evaluated by pediatric endocrinologists and pediatric radiologists at a tertiary care center between December 2021 and December 2023.

### 2.1. Laboratory Tests

Routine diagnostic blood and urine tests for T1DM were conducted in the patient group. These included measurements of glucose, ketones, insulin, C-peptide, HbA1c, pH, and HCO_3_ levels. Additionally, autoantibody tests were performed, including Anti-GAD, Islet Cell Antibody, and Insulin Antibody. No additional laboratory tests were performed in the control group. Clinical and laboratory data from the patients with T1DM were collected for statistical analysis.

### 2.2. Pancreas Ultrasonography: Pancreatic Size and Echogenicity

**Figure 1:**
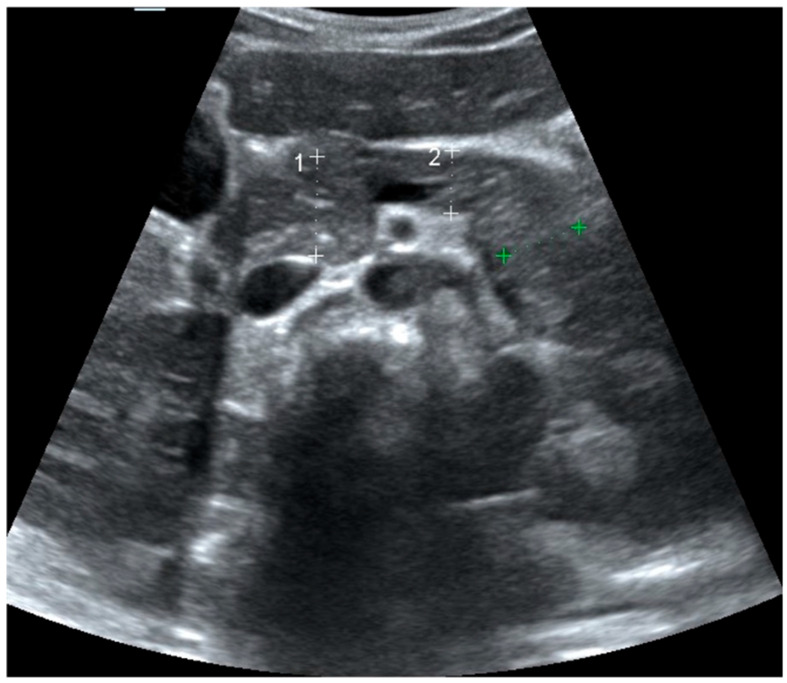
Upper abdominal ultrasound. Pancreatic size measurements: 1 = head, 2 = body; tail shown in green. For each region, the measurement corresponds to the dotted line between the two “+” caliper marks—white “+” for head (1) and body (2), green “+” for tail.

Ultrasonographic evaluations of the pancreas were performed within five days of T1DM diagnosis in metabolically stable patients who had initiated short- and long-acting conventional insulin treatment. Visualisations were performed by one experienced pediatric radiologist under the supervision of a senior pediatric radiologist using the LOGIQ S8 ultrasound system (General Electric Medical Systems, Milwaukee, WI, USA) equipped with a convex array transducer (1–6 MHz). To ensure reproducibility, all ultrasonographic assessments were performed using standardized scanning protocols under fasting conditions and with identical equipment and transducer settings. Measurements were obtained three times for each pancreatic segment (head, body, and tail), and the mean values were used for analysis to minimize intra-observer variability. Established pediatric pancreatic USG protocols were followed to ensure reliability [8,9]. A pancreatic image from a single participant, illustrating the head, body, and tail measurement locations, is presented in Figure 1.

**Figure 2:**
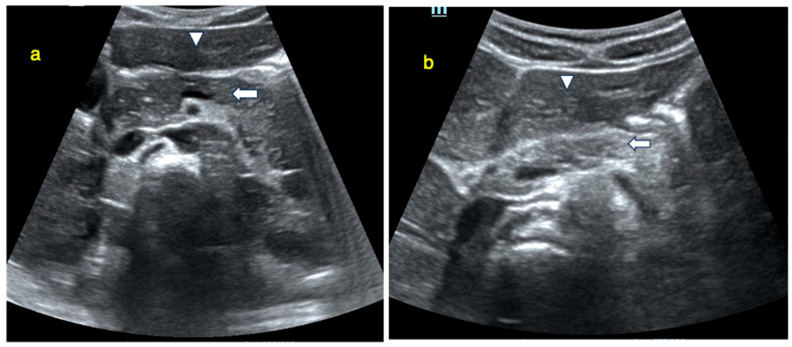
Ultrasound image showing pancreas echogenity compared to liver. (**a**): Ultrasound image showing pancreas (arrow) with equal echogenity compared to liver (arrowhead). (**b**): Ultrasound image showing hyperechoic pancreas (arrow) compared to liver (arrowhead).

An isoechoic pancreas is defined as having an echo intensity equal to or slightly higher than that of the liver. Echogenicity was assessed by comparing the pancreas to the adjacent liver at similar imaging depths and categorized as isoechogenic or non-isoechogenic (hypoechogenic or hyperechogenic). This comparative assessment between the pancreas and liver is a standard approach in radiological imaging [9,10]. Pancreatic images from participants illustrating echogenicity patterns—isoechogenic (panel a) versus non-isoechogenic (panel b)—are presented in Figure 2. Alterations in echogenicity, such as areas of hypo- or hyperechogenicity, may indicate pathological conditions such as acute or chronic pancreatitis [11]. To enhance pancreatic visualization, patients fasted for at least six hours to reduce intestinal gas and peristaltic motion. Additionally, 200 mL of water was administered before the examination to improve pancreatic delineation during scanning [12].

### 2.3. Statistical Analysis

Statistical analyses were conducted using SPSS (Statistical Package for Social Sciences; SPSS Inc., Chicago, IL, USA) version 22. Descriptive data in the study are presented as “*n*” and “%” values for categorical data, and as “mean ± standard deviation” (Mean ± SD) for continuous data. The Chi-square test (Pearson Chi-square) was applied to compare categorical variables between groups. The Kolmogorov–Smirnov test was used to assess the normality of the distribution of continuous variables. Student’s *t*-test was used for comparisons of two groups, and One Way ANOVA was used for comparisons involving more than two variables. The Pearson correlation test was employed to examine the relationships between continuous variables. A *p*-value of “<0.05” was considered statistically significant in the analyses.

## 3. Results

Comparison of Demographic and Ultrasonographic Findings Between Newly Diagnosed T1DM Patients and Healthy Controls: A total of 147 children were included in the study, consisting of 69 patients with newly diagnosed T1DM and 78 healthy controls. Among the patients, 27.5% were female and 72.5% were male, while in the control group, 30.8% were female and 69.2% were male. There was no significant difference between the groups in terms of sex (*p* = 0.667). The mean age of the patient group was 9.9 ± 4.7 years, and the mean age of the control group was 10.0 ± 4.6 years, with no significant difference between the groups (*p* = 0.894). Measurements of the pancreatic head, body, and tail were significantly smaller in the patients with newly diagnosed T1DM compared to the control group (*p* < 0.001). A comparison of the demographic and ultrasonographic characteristics of both groups is presented in Table 1.

Relationship of Pancreatic Echogenicity With Clinical Parameters in T1DM Patients: In the patient group, the mean age of patients with non-isoechogenic pancreas on USG was significantly higher compared to those with isoechoic pancreas on USG (*p* = 0.005). The mean BMI SDS at diagnosis was similar between the groups, with 0.01 ± 1.02 in the isoechoic group and 0.01 ± 1.28 in the non-isoechogenic group (*p* = 0.978). Among the patients, a family history of type 1 diabetes was reported in 5 cases. Isoechoic echogenicity was observed in 60.0% of these patients, while 40.0% had non-isoechogenic findings, with no significant difference compared to those without a family history (*p* = 0.967). A comparison of pancreatic echogenicity with clinical parameters is presented in Table 2.

Age-Related Differences in Pancreatic Echogenicity, Size Reduction, and Clinical Parameters in Children With Newly Diagnosed T1DM: Patients were divided into three age groups (0–6, 7–12, and 13–18 years), and their clinical and laboratory data were analyzed.

The analysis revealed that 80% of patients in the 0–6 age group, 63% in the 7–12 age group, and 40.9% in the >13 age group had an isoechoic pancreas, with a statistically significant difference between the groups (*p* = 0.033). The rate of pancreatic size reduction was compared between patients under 7 years old (early childhood) and healthy controls, as well as between teenage patients and healthy controls. In patients under 7 years old (*n* = 20), the reduction rates in the pancreatic head and body sizes were 14.4% and 15.5%, respectively, whereas in patients over 13 years old (*n* = 22), these rates were notably higher, at 21% and 26.7%, respectively. Additionally, significant differences were observed in C-peptide (*p* = 0.002) and HbA1c levels (*p* = 0.048) among the age groups. These differences were mainly attributed to the variation between the <7 age group and the >13 age group. The mean BMI SDS did not differ significantly across age groups, with values of 0.3 ± 1.0 in children aged 0–6 years, 0.0 ± 1.2 in those aged 7–12 years, and −0.2 ± 1.1 in adolescents ≥13 years (*p* = 0.454). A family history of type 1 diabetes was reported in 1 patient (5.0%) in the 0–6 years group, 2 patients (7.4%) in the 7–12 years group, and 2 patients (9.1%) in the ≥13 years group, with no significant difference between groups (*p* = 0.877). Further clinical analyses of the patients, subcategorized by age, are presented in Table 3.

Demographic and Clinical Profile of Patients With Type 1 Diabetes Mellitus: In this study, 27.5% of the patients with Type 1 DM were female and 72.5% were male, with a mean age of 9.9 ± 4.7 years (range: 1.2–17.8 years). A family history of Type 1 DM was present in 7.2% of the patients. Additional clinical characteristics of the patients are summarized in Table 4.

## 4. Discussion

In this prospective case–control study, we conducted the first ultrasonographic evaluation of pancreatic morphology in pediatric patients with newly diagnosed T1DM within five days of diagnosis. The findings revealed significant age-related differences in pancreatic size and echogenicity that align with established immunological endotypes. Compared to healthy controls, T1DM patients exhibited marked pancreatic atrophy across dimensions (*p* < 0.001), with greater reductions in adolescents (≥13 years; head: 21%, body: 26.7%) than in the younger age group (<7 years; head: 14.4%, body: 15.5%). Additionally, isoecogenicity predominated in younger patients (80% vs. 40.9% in adolescents; *p* = 0.033), while heterogeneous echogenicity was more common in older groups. These structural changes correlated with clinical parameters, including higher C-peptide and HbA1c levels in adolescents, supporting the hypothesis of distinct endotypic mechanisms in pediatric T1DM.

Our study revealed a marked reduction in pancreatic size in pediatric patients at the time of T1DM diagnosis. Although beta cells constitute only about 2% of the pancreas, several mechanisms may underlie this atrophy. First, inflammation in the exocrine tissue, accompanied by the infiltration of white blood cells and the presence of autoantibodies contributes to pancreatic atrophy and a reduction in acinar cell numbers [13]. Second, the loss of insulin’s trophic effects may exacerbate the reduction in glandular mass [14]. A decrease in pancreatic volume is a known feature of T1DM. Notably, reduced pancreatic volume has been reported in certain at-risk populations even before the clinical manifestation of T1DM. Studies have shown that antibody-positive relatives without diabetes of individuals with T1DM exhibit reduced pancreatic volumes [15]. In patients, this reduction continues to progress following diagnosis. In a longitudinal study by Wright et al. involving 91 T1DM patients and controls, pancreatic volumes decreased within the first year after diagnosis and continued to decline over a 1–5-year period [16]. These findings are aligned with the study by Evans-Molina et al., which described beta-cell decline beginning up to five years prior to clinical diagnosis [17]. Similarly, Virostko et al.’s longitudinal study, involving 51 pediatric T1DM patients and 57 controls, confirmed reduced pancreatic size in children with diabetes following diagnosis using MRI [18]. Unlike Virostko et al.’s study, which utilized MRI an average of 67 days post-diagnosis, imaging in our study was performed soon after patients reached metabolic stability and started both short- and long-acting conventional insulin therapy. USG evaluations revealed structural changes in size and echogenicity at the initial recognition of the disease. These findings show that use of USG at such an early stage represents a novel approach, capturing preclinical changes before extensive fibrosis or adaptation. Unlike MRI/CT, which are resource-intensive, USG offers accessibility and real-time assessment, making it suitable for pediatric settings.

Our study found increased non-isoechogenicity accompanied by reduced pancreatic size in both early childhood and adolescent patients with T1DM compared to healthy controls. These changes were more significant in the adolescent group than in the early childhood group. This age-related difference aligns with the endotype models of pediatric-onset T1DM, which presents distinct presentations and progression patterns across age groups: in children under the age of 7, the disease is typically classified as type 1 diabetes endotype-1 (T1DE1), characterized by rapid and aggressive inflammation. In contrast, in those older than 13 years, it follows a slower disease course, which is classified as endotype 2 (T1DE2). T1DE1 is driven by IFNγ-mediated rapid inflammation, whereas T1DE2 is characterized by IL-10-modulated immune responses, leading to a slower but prolonged course of pancreatic damage that begins before the onset of clinical symptoms [19,20]. These findings align with Achenbach et al., who identified distinct immunological profiles in 1192 individuals across different age groups [21]. Similarly, Leete et al.’s histological data revealed high-CD20 profiles in younger T1DM patients and low-CD20 profiles in older patients, supporting endotypic immunological differences in T1DM [22]. In our cohort, the 7–12 year age group exhibited intermediate values across clinical and immunological parameters. Rather than indicating a separate endotype, these findings suggest that this group may represent a transitional phenotype, sharing characteristics of both early-onset (<7 years) and adolescent-onset (≥13 years) T1DM. This mixed pattern supports the idea that disease heterogeneity changes with age. Our findings suggest that age-specific immune endotypes in T1DM may affect pancreatic structure differently. The more pronounced changes in adolescents may reflect the slower course of T1DE2, allowing more damage to occur before diagnosis. This also suggests that preclinical disease progression may vary by age.

In addition to structural differences, laboratory features at diagnosis also varied by age. Early childhood T1DM patients (0–6 years) exhibited significantly lower C-peptide levels and higher ketone and at diagnosis compared to adolescents (≥13 years) in our study group. Similarly, Leete et al. defined T1DE1 patients as having histologically high proinsulin:C-peptide ratios and intense immune activity (CD20Hi), whereas T1DE2 patients (diagnosed at ≥13 years) exhibited a milder immune response and greater preservation of C-peptide. In their long-term follow-up, individuals diagnosed at ≥13 years showed significantly higher stimulated C-peptide levels compared to those diagnosed before the age of 7 [22]. These findings may be attributed to the more aggressive immune activation observed in T1DE1 phenotypes, as noted above. However, Virotsko et al. demonstrated that patients newly diagnosed with T1DM (median age 13 years) showed no correlation between C-peptide levels and pancreatic volume (*p* = 0.5), suggesting that exocrine atrophy may not directly parallel beta-cell dysfunction [18]. This may be explained by the predominance of adolescent, T1DE2-like phenotypes in their cohort. In contrast, our cohort included a more balanced age distribution (mean age 9.9 ± 4.7 years), with approximately one-third of the patients under 7 years and one-third over 13 years. This allowed us to capture both T1DE1 and T1DE2 phenotypes and differentiate between them. Leete et al.’s proinsulin:C-peptide ratio could serve as a marker for spotting T1DE1 in younger patients, complementing Virotsko et al.’s MRI measurements. Further research is needed to investigate immune differences, particularly the role of inflammation in T1DE1, as age-related immune patterns are not well-studied.

No significant association was found between the presence, type, or levels of diabetes-related autoantibodies and pancreatic heterogeneity at T1DM diagnosis, even in patients exhibiting multiple autoantibody positivity. However, anti-GAD positivity was significantly more common in adolescents compared to younger children. This age-related pattern is consistent with findings from Tridgell et al., who analyzed 5020 subjects from the T1DM Genetic Database and reported a higher prevalence of anti-GAD antibodies in individuals diagnosed after the age of 13 [23]. Similarly, Herold et al. observed that the initial autoantibody profile varies with age, likely influenced by genetic predisposition and HLA class subtypes. They also highlighted the critical role of antigen-presenting β-cells in T1DM pathogenesis, suggesting that while autoantibodies are strongly associated with disease onset, they may play a secondary role relative to β-cell-driven antigen presentation mechanisms [5]. The absence of correlation between autoantibodies and pancreatic echogenicity in our cohort may have several explanations. First, the modest sample size limited the statistical power, particularly when subdividing patients by individual antibody type. Second, autoantibodies mainly reflect immune activity directed against β-cells, whereas changes in echogenicity are thought to represent chronic alterations of the exocrine pancreas such as inflammatory infiltration, acinar loss, or fibrosis, which may occur independently of antibody status [24,25]. Third, these processes likely follow different temporal patterns: autoantibodies may appear long before clinical onset, whereas structural changes in the exocrine pancreas may develop closer to diagnosis and continue to progress thereafter. Previous studies have also highlighted this discordance. In some individuals, exocrine involvement appears before autoantibody seroconversion, whereas in others, parenchymal changes occur independently of antibody profiles. Although autoantibodies are strong predictors of disease progression, their association with exocrine remodeling remains modest and inconsistent [24,25]. Collectively, our findings suggest that while autoantibodies remain essential for the diagnosis and prediction of T1DM, they do not appear to account for the structural heterogeneity of the pancreas at onset. This suggests that echogenicity may reflect additional, antibody-independent mechanisms contributing to parenchymal involvement in T1DM.

Notably, increased pancreatic echogenicity in our cohort did not correlate with clinical or biochemical markers of disease severity at presentation (e.g., pH, HbA1c, ketone, or C-peptide levels). The lack of correlation between echogenicity and clinical or laboratory severity at presentation supports the view that echogenicity reflects chronic, rather than acute, disease processes. These results are consistent with previous studies showing progressive increases in pancreatic echogenicity over time following diagnosis [26].

In our cohort, most patients were male (72.5%). Although some studies have reported a slight male predominance and others found no significant sex difference, the relatively high proportion of male patients in our study should be interpreted with caution, given the limited sample size and single-center design, which may have influenced the observed sex distribution [27].

This study has several limitations. As a single-center investigation with a modest sample size (*n* = 69), generalizability may be constrained, particularly regarding ethnic diversity or environmental factors. The absence of genetic profiling (e.g., HLA typing) or longitudinal follow-up limits inferences on causality and progression. The absence of laboratory tests (e.g., fasting glucose, C-peptide) in controls, to minimize invasive procedures in healthy children, represents a limitation. Additionally, USG’s operator-dependency and lack of volumetric quantification (unlike MRI) could introduce variability, though standardized protocols mitigated this. In our study, two pediatric radiologists were involved: measurements were performed by a single radiologist under the supervision of a senior radiologist with over 20 years of experience. The control group was age- and sex-matched at a 1:1 ratio to maximize statistical power and minimize sampling bias within the prospective design. Therefore, the potential impact of operator dependency on our results is expected to be minimal. Future research should encompass multi-center cohorts, incorporate advanced imaging (e.g., contrast-enhanced USG), and track long-term outcomes to validate USG’s prognostic value. Exploring correlations with insulitis biomarkers or endotype-specific therapies would further elucidate mechanisms. Despite these limitations, this study has several notable strengths. The inclusion of a wide age range (mean 9.9 ± 4.7 years) enabled the analysis of both early childhood (T1DE1) and adolescent (T1DE2) endotypes, revealing age-related differences in pancreatic structure and clinical parameters. The case–control design, with age- and sex-matched healthy controls, strengthened the detection of disease specific changes. Ultrasonographic evaluations were performed by experienced pediatric radiologists using standardized protocols and a high-quality ultrasound system, providing reliable and reproducible measurements. The study’s prospective, cross-sectional design enabled early evaluation of pancreatic changes at T1DM diagnosis.

## 5. Conclusions

Pancreatic structural changes at T1DM onset are heterogeneous across childhood, reflecting underlying immunological endotypes. Pancreatic remodeling appears to follow distinct age-related immune patterns, linking endocrine and exocrine compartments within a single disease process. Beyond its diagnostic utility, USG—being noninvasive, cost-effective, and easily repeatable—may serve as a practical tool for screening at-risk children, particularly those with autoantibody positivity or genetic susceptibility. Serial, non-invasive USG assessments may also guide decisions regarding immunomodulatory therapies (e.g., teplizumab) and facilitate monitoring of treatment responses.

## Figures and Tables

**Table 1:** Comparison of demographic and ultrasonography features of the groups.

		Patient (*n* = 69)		Control (*n* = 78)		*p*
		*n*	%	*n*	%	
Sex	Female	19	27.5	24	30.8	0.667 *
	Male	50	72.5	54	69.2	
Age (years)		9.9 ± 4.7 (8.8–11.0)		10.0 ± 4.6 (9.0–11.0)		0.894 **
Pancreatic head (mm)		11.6 ± 2.4 (11.0–12.2)		14.4 ± 2.5 (13.8–15.0)		<0.001 **
Pancreatic body (mm)		9.1 ± 1.7 (8.7–9.5)		12.0 ± 2.1 (11.5–12.5)		<0.001 **
Pancreatic tail (mm)		9.9 ± 1.9 (9.4–10.4)		12.4 ± 2.0 (11.9–12.9)		<0.001 **
Pancreatic echogenicity (compared to liver)	Isoechoic	42	60.9	60	76.9	0.035 *
	Hypo/Hyperechoic	27	39.1	18	23.1	

* Chi-square test, ** Student’s *t*-test used.

**Table 2:** Comparison of pancreatic echogenicity of the patients based on clinical parameters.

		Isoechoic		Non-Isoechogenic		*p* *
Patient Group (*n*:69)		*n*	%	*n*	%	
Sex	Female	11	57.9	8	42.1	0.755
	Male	31	62.0	19	38.0	
Age, years, Mean ± SD		8.6 ± 4.5 (7.2–10.0)		11.8 ± 4.5 (10.1–13.5)		0.005 **
Diagnosis Glucose, mg/dL, Mean ± SD		463.9 ± 149.9 (417.2–510.6)		473.4 ± 149.8 (414.1–532.7)		0.798 **
Diagnosis Insulin, Mean ± SD		2.9 ± 4.5 (1.5–4.3)		2.9 ± 2.0 (2.1–3.7)		0.996 **
Diagnosis C-peptide, µg/L, Mean ± SD		0.5 ± 0.3 (0.4–0.6)		0.6 ± 0.4 (0.4–0.8)		0.080 **
Diagnosis HgbA1C, Mean ± SD		11.8 ± 2.3% (11.1–12.5)		12.2 ± 1.7% (11.5–12.9)		0.368 **
Diagnosis pH, Mean ± SD		7.3 ± 0.2 (7.2–7.4)		7.3 ± 0.1 (7.3–7.3)		0.568 **
Diagnosis HCO_3_, mmol/L, Mean ± SD		15.0 ± 7.2 (12.8–17.2)		15.5 ± 7.7 (12.5–18.5)		0.810 **
Diagnosis Ketone, mmol/L, Mean ± SD		4.0 ± 2.1 (3.3–4.7)		3.3 ± 2.0 (2.5–4.1)		0.202 **
Anti GAD	Positive	24	60.0	16	40.0	0.953
	Negative	17	60.7	11	39.3	
Anti-GAD Level IU/mL, Mean ± SD		76.7 ± 95.6 (46.9–106.5)		48.1 ± 61.8 (23.7–72.5)		0.139 **
Islet Cell Antibody	Positive	22	64.7	12	35.3	0.457
	Negative	19	55.9	15	44.1	
Islet Cell Antibody Level U/mL, Mean ± SD		61.2 ± 76.0 (37.5–84.9)		46.5 ± 57.7 (23.7–69.3)		0.396 **
Insulin Antibody	Positive	6	85.7	1	14.3	0.223
	Negative	31	54.4	26	45.6	
Insulin Antibody Level, Mean ± SD		6.1 ± 5.0% (4.5–7.7)		5.3 ± 4.2% (3.6–7.0)		0.465 **
Number of Antibody Positivity	None	8	44.4	10	55.6	0.506
	1	13	68.4	6	31.6	
	2	15	60.0	10	40.0	
	3	1	50.0	1	50.0	

* Chi-square test, ** Student’s *t*-test used.

**Table 3:** Clinical Characteristics and Analyses of Patients Grouped by Age.

		0–6 Year Olds		7–12 Year Olds		≥13 Year Olds		*p*
		*n*	%	*n*	%	*n*	%	
Sex	Female	3	15.0	12	44.4	4	18.2	0.041 *
	Male	17	85.0	15	55.6	18	81.8	
Pancreatic echogenicity	Isoechoic	16	80.0 ^a^	17	63.0 ^a,b^	9	40.9 ^b^	0.033 *
	Hypo/Hyperechoic	4	20.0	10	37.0	13	59.1	
Diagnosis Glucose, mg/dL, Mean ± SD		473.3 ± 92.9 (428–518)		472.9 ± 177.8 (406–543)		456.0 ± 156.7 (386–525)		0.909 **
Diagnosis Insulin, Mean ± SD		3.1 ± 6.4 (0.1–6.1)		2.8 ± 1.8 (2.1–3.5)		3.0 ± 1.6 (2.3–3.7)		0.981 **
Diagnosis C-peptide, µg/L, Mean ± SD		0.4 ± 0.2 ^a^ (0.3–0.5)		0.6 ± 0.3 ^a,b^ (0.5–0.7)		0.7 ± 0.3 ^b^ (0.6–0.8)		0.002 **
Diagnosis HgbA1C, Mean ± SD		11.1 ± 1.9 ^a^ (10.2–12.0)		11.9 ± 2.0 ^a,b^ (11.1–12.7)		12.7 ± 2.1 ^b^ (11.8–13.6)		0.048 **
Diagnosis pH, Mean ± SD		7.3 ± 0.1 (7.3–7.3)		7.3 ± 0.2 (7.2–7.4)		7.3 ± 0.1 (7.3–7.3)		0.519 **
Diagnosis HCO_3_, mmol/L, Mean ± SD		12.4 ± 7.0 (9.1–15.7)		16.8 ± 6.9 (14.1–19.5)		15.9 ± 7.8 (12.4–19.4)		0.117 **
Diagnosis Ketone, mmol/L, Mean ± SD		4.7 ± 1.8 ^a^ (3.9–5.5)		3.0 ± 2.0 ^b^ (2.2–3.8)		3.7 ± 2.1 ^a,b^ (2.8–4.6)		0.035 **
Anti GAD	Positive	7	36.8 ^a^	16	59.3 ^a,b^	17	77.3 ^b^	0.032 *
	Negative	12	63.2	11	40.7	5	22.7	
Islet Cell Antibody	Positive	6	31.6	17	63.0	11	50.0	0.111 *
	Negative	13	68.4	10	37.0	11	50.0	
Insulin Antibody	Positive	6	31.6 ^a^	0	0.0 ^b^	1	4.8 ^a,b^	0.002 *
	Negative	13	68.4	24	100.0	20	95.2	
Anti-GAD Level IU/mL, Mean ± SD		44.0 ± 65.0 (13.6–74.4)		75.3 ± 97.4 (36.6–113.8)		71.6 ± 82.6 (35.0–108.2)		0.432 **
Islet Cell Antibody Level U/mL, Mean ± SD		28.9 ± 41.8 (9.3–48.5)		67.9 ± 77.6 (37.2–98.6)		62.9 ± 73.6 (30.3–95.5)		0.140 **
Insulin Antibody Level, Mean ± SD		7.9 ± 6.4 ^a^ (4.9–10.9)		4.6 ± 1.6 ^b^ (4.0–5.2)		5.2 ± 4.8 ^a,b^ (3.1–7.3)		0.045 **
Number of Antibody Positivity	0	9	47.4	5	20.8	4	19.0	0.160 *
	1	2	10.5	9	37.5	8	38.1	
	2	7	36.8	10	41.7	8	38.1	
	3	1	5.3	0	0.0	1	4.8	

* Chi-square test, ** One Way ANOVA analysis used. Superscript letters (a,b) indicate results of pairwise comparisons among age groups within the same row. Groups that do not share a letter (e.g., a vs. b) are significantly different at α = 0.05; groups that share at least one letter (e.g., a and a,b) are not significantly different.

**Table 4:** Characteristics of Patients with Type 1 DM.

Symptom Duration (Days), Mean ± SD		24.5 ± 23.2 (18.9–30.1)	
BMI SDS, Mean ± SD		0.01 ± 1.12 (−0.3–0.3)	
Dehydration Severity	Mild	4	5.8
	Moderate	51	73.9
	Severe	6	8.7
	None	8	11.6
Initial Diagnosis	Hyperglycemia	26	37.7
	Ketosis	16	23.2
	Ketoacidosis	27	39.1
DKA Severity	Mild	8	29.6
	Moderate	7	25.9
	Severe	12	44.4
Diagnosis Glucose, mg/dL, Mean ± SD		467.6 ± 148.8 (431.9–503.3)	
Diagnosis Ketone, mmol/L, Mean ± SD		3.8 ± 2.1 (3.3–4.3)	
Diagnosis Insulin, Mean ± SD		2.9 ± 3.7 (2.0–3.8)	
Diagnosis C-peptide, µg/L, Mean ± SD		0.5 ± 0.3 (0.4–0.6)	
Diagnosis HgbA1C, Mean ± SD		11.9 ± 2.1% (11.4–12.4)	
Diagnosis pH, Mean ± SD		7.3 ± 0.1 (7.3–7.3)	
Diagnosis HCO_3_, mmol/L, Mean ± SD		15.2 ± 7.3 (13.4–17.0)	
Anti GAD	Positive	40	58.8
	Negative	28	41.2
Anti-GAD Level (IU/mL), Mean ± SD		65.4 ± 84.5 (45.1–85.7)	
Islet Cell Antibody	Positive	34	50.0
	Negative	34	50.0
Islet Cell Antibody Level U/mL, Mean ± SD		55.4 ± 69.2 (38.8–72.0)	
Insulin Antibody	Positive	7	10.9
	Negative	57	89.1
Insulin Antibody Level, Mean ± SD		5.8 ± 4.7% (4.7–6.9)	

## Data Availability

The data are available from the corresponding author upon reasonable request.

## Abbreviations

Anti-GAD	Anti-Glutamic Acid Decarboxylase (antibody)
BMI SDS	Body Mass Index Standard Deviation Score
CT	Computed Tomography
DKA	Diabetic Ketoacidosis
HbA1c	Hemoglobin A1c
HCO_3_	Bicarbonate
IFNγ	Interferon Gamma
IL-10	Interleukin-10
MRI	Magnetic Resonance Imaging
SD	Standard Deviation
SPSS	Statistical Package for the Social Sciences
T1DE1	Type 1 Diabetes Endotype 1 (early childhood onset, <7 years)
T1DE2	Type 1 Diabetes Endotype 2 (adolescent onset, ≥13 years)
T1DM	Type 1 Diabetes Mellitus
USG	Ultrasonography
